# Research Trends and Emerging Hotspots of Lung Cancer Surgery during 2012-2021: A 10-Year Bibliometric and Network Analysis

**DOI:** 10.34133/2022/9797842

**Published:** 2022-10-20

**Authors:** Jingyi Wu, Chenlu Bao, Ganwei Liu, Shushi Meng, Yunwei Lu, Pengfei Li, Jian Zhou

**Affiliations:** ^1^Advanced Institute of Information Technology, Peking University, Hangzhou 311215, China; ^2^Department of Thoracic Surgery, Peking University People’s Hospital, Beijing 100044, China; ^3^Department of Thoracic Surgery, Beijing Haidian Hospital, Beijing 100000, China; ^4^National Institute of Health Data Science, Peking University, Beijing 100191, China

## Abstract

*Background*. Lung cancer remains the leading cause of death because of cancer globally in the past years. To inspire researchers with new targets and path-breaking directions for lung cancer research, this study is aimed at exploring the research trends and emerging hotspots in the lung cancer surgery literature in the recent decade.*Methods*. This cross-sectional study combined bibliometric and network analysis techniques to undertake a quantitative analysis of lung cancer surgery literature. Dimensions database was searched using keywords in a 10-year period (2012-2021). Publications were characterized by publication year, research countries, field citation ratio, cooperation status, research area, and emerging hotspots.*Results*. Overall, global scholarly outputs of lung cancer surgery had almost doubled during the recent decade, with China, Japan, and the United States leading the way, while Denmark and Belgium predominated in terms of scientific influence. Network analysis showed that international cooperation accounted for a relatively small portion in lung cancer surgery research, and the United States, China, and Europe were the prominent centers of international cooperation network. In the recent decade, research of lung cancer surgery majored in prevention, biomedical imaging, rehabilitation, and genetics, and the emerging research hotspots transformed into immunotherapy. Research on immunotherapy showed a considerable increase in scientific influence in the latest year.*Conclusions*. The study findings are expected to provide researchers and policymakers with interesting insights into the changing trends of lung cancer surgery research and further generate evidence to support decision-making in improving prognosis for patients with lung cancer.

## 1. Introduction

According to the latest global cancer statistic research, an estimated 2.2 million newly diagnosed cases and 1.8 million death cases caused by lung cancer were reported in 185 countries in 2020 [1]. Lung cancer remains the leading cause of death because of cancer globally in the past few decades [2]. With raising awareness of the influence of lung cancer, an increasing number of studies related to lung cancer were published every year [3, 4]. More specifically, over 240,000 publications between 1975 and 2019 could be accessed from the Web of Science database based on the keyword “lung cancer” [5]. Thoracic surgery is acknowledged as the standard treatment for primary lung cancer [6, 7]. In the past decades, evolution of video-assisted thoracic surgery (VATS) significantly decreases trauma size and improves survival of patients with lung cancer [8]. This furtherly increased the research enthusiasm on thoracic surgery in lung cancer. The scholarly output on thoracic surgery published between 2004 and 2013 were ranked at 4 among subcategorized topics of lung cancer research [4]. Using keywords related to lung cancer surgery, over 18,000 publications between 1945 and 2018 could be accessed through the Web of Science database [9].

The rapidly increasing number of publications related to lung cancer surgery makes it more and more difficult for researchers to keep up with the latest findings, even inside their domain of expertise. The comprehensive information of a specific research field, such as the quantitative growth trend, the distribution of scientific influence, cooperation status of countries, and emerging trends of future research hotspots, are meaningful and inspirational for researchers. Reporting the changing trends and hotspots of thoracic surgery research in lung cancer could potentially inspire researchers to identify new targets and path-breaking directions for research and generate evidence for policymakers to support decision-making. Furthermore, this could help achieve better treatment and prognosis in patients with lung cancer.

Bibliometric analysis is an effective method to systemically and quantitatively identify knowledge structure and evaluate research trends of a specific field from multiple dimensions. To better understand and keep track of the changing landscape of research related to lung cancer surgery in the recent decade, this study combined the bibliometric and network analysis techniques to conduct a quantitative assessment of the research trends and hotspots of lung cancer surgery. Using data from the Dimensions database, the research trends of scholarly output and scientific influence, cooperation network, and emerging hotspots in lung cancer surgery research were analyzed. We aimed to provide researchers and policymakers with interesting insights into the changing research trends in the field of lung cancer surgery and further help achieve a better prognosis for patients with lung cancer. Three main hypotheses are proposed in this study. First, echoing on the increasing burden of lung cancer, there is a substantial increase in global scholarly outputs of lung cancer surgery research during the recent decade, with a diverse distribution across countries. Second, resembling findings of cooperation networks in other contexts like cancer and cardiovascular diseases [10, 11], the United States and Europe are the prominent centers of international cooperation network in the field of lung cancer surgery. Third, consistent with the research trend in the field of lung cancer [4, 12, 13], there is an increasing output in basic science research while a decreasing output in clinical translational research in the field of lung cancer surgery, and immunotherapy and neoadjuvant therapy are emerging research hotspots.

## 2. Methods

To map the scientific landscape related to lung cancer surgery from 2012 to 2021, we conducted bibliometric and network analysis techniques to analyze scientific publication data available in the Dimensions database. Developed by the Digital Science in collaboration with over 100 leading research organizations around the world, Dimensions is an openly available, comprehensive research knowledge database that covers data of grants, publications, citations, alternative metrics, clinical trials, and patents. Dimensions has covered over 129 million publications, indexed via Crossref, PubMed, PubMed Central, arXiv, and more than 160 publishers directly. Dimensions has about 25% more publication records than other databases like Google Scholar, PubMed, Scopus, and Web of Science. Besides, using machine learning and cloud computing technology, Dimensions standardized the data based on a common data model before data integration and derived a series of bibliometric indicators for data enrichment. For example, the information of researchers and organizations of publications is standardly indexed and disambiguated, which could ensure the accuracy of bibliometric analysis, and rich indicators ranging from research areas and concepts to impact indicators based on citations and Altmetric attention were algorithmically extracted for comprehensive research. Therefore, Dimensions database could present a broader scientific landscape. Publication records in Dimensions are updated daily. Related citation metrics are updated either daily or 2-4 times a year. Further details about the Dimensions database are available in the published articles [14, 15] or the official website (https://www.dimensions.ai/).

### 2.1. Ethics Statement

The data used in this study are publicly available and contain no protected health information. Therefore, approval of the Ethics Committee of Peking University People’s Hospital was not sought. This study followed the Strengthening the Reporting of Observational Studies in Epidemiology (STROBE) reporting guideline for cross-sectional studies.

### 2.2. Search Strategy and Eligibility Criteria

**Figure 1:**
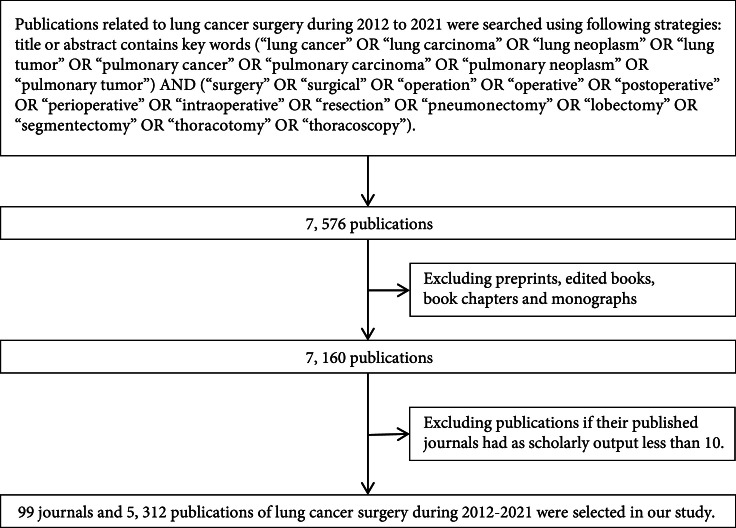
Flowchart of publications selection in this study.

Flowchart of publication selection in this study is shown in Figure 1. We used the following search strategies in Dimensions to identify scientific publications related to lung cancer surgery from 2012 to 2021 referring to the existing literature [9, 16, 17]: title or abstract contains keywords (“lung cancer” or “lung carcinoma” or “lung neoplasm” or “lung tumor” or “pulmonary cancer” or “pulmonary carcinoma” or “pulmonary neoplasm” or “pulmonary tumor”) and (“surgery” or “surgical” or “operation” or “operative” or “postoperative” or “perioperative” or “intraoperative” or “resection” or “pneumonectomy” or “lobectomy” or “segmentectomy” or “thoracotomy” or “thoracoscopy”). The search obtained 7,576 records during the study period (2012-2021). Publications in Dimensions consist of journal articles, preprints, edited books, book chapters, and monographs. Only journal articles were included in our study because they are more time-sensitive. To ensure the quality and representativeness of publications for analysis in the field of lung cancer surgery research, we excluded publications if their published journals had a scholarly output less than 10. Finally, a total of 99 journals and 5,312 publications of lung cancer surgery during 2012-2021 were selected in our study. 

### 2.3. Data Extraction

For each publication of lung cancer surgery, the following data were extracted from Dimensions: year of publication, field citation ratio (FCR), countries of researchers, research organizations, cooperation type, research area, and research concepts. FCR is a citation-based measure of scientific influence of publications and was calculated by dividing the number of citations a publication has received by the average number received by articles published in the same year and Fields of Research (FoR) category [18, 19]. For example, a publication with an FCR of 2.0 has received twice as much scientific attention as the average of its research field. Compared with raw count of citations, FCR is a more accurate scientific influence measure of publications because it has been normalized both for research field and year of publication [20]. Cooperation type of a publication was identified as (1) institutional, if all its authors come from one organization, (2) national, if its authors come from two or more organizations in the same country, or (3) international, if its authors come from two or more countries. 

The research area of each publication was categorized using the Research, Condition, and Disease Categorization (RCDC) system developed by the National Institutes of Health of United States [21]. Dimensions identified RCDC category of each publication automatically by machine learning. In this study, only RCDC categories related to research fields were included. A series of research concepts, describing the main topics of a publication, were automatically derived from the publication text (including title, keywords, and abstract) via machine learning methods as well, and a very good match was achieved compared to manual coding (Supplementary Materials). 

### 2.4. Data analysis

This study is aimed at presenting the research trends and emerging hotspots of lung cancer surgery in the recent decade. We analyzed the trends of scholarly outputs and scientific influence (FCR) of publications related to lung cancer surgery from both temporally and spatially. The possible associations of the research trends and the disease burden of lung cancer were identified as well. Disease burden of lung cancer in different countries was measured with the data obtained from the Global Burden of Disease Study 2019 (GBD 2019) [2] using disability-adjusted life year (DALY). 

A global cooperation network in lung cancer surgery research was developed as well. In the cooperation network, a point refers to a country, and its size describes numbers of cooperation partnerships of this country; a line refers to a cooperation link between countries, and the link strength describes numbers of publications under cooperation of the two countries.

The emerging research hotspots in the field of lung cancer surgery in the recent 10 years were also reported in this study to investigate new research topics in the field of lung cancer surgery in recent years. A research concept was identified as emerging in a year if it did not emerge in the selected publications until this year. Top five frequent emerging research concepts from 2012 to 2021 were identified as emerging research hotspots during this period. These emerging research hotspots were categorized into different Medical Subject Headings (MeSH) system categories by experienced clinicians based on the MeSH on Demand tool (https://meshb.nlm.nih.gov/MeSHonDemand) developed by the National Library of Medicine, United States. The average FCRs of each hotspot concept in the emerging year, the latest year, and the recent 10 years were reported to demonstrate the scientific influence trends of the emerging research topics. 

All the data analyses were conducted using Python (version 3.8.5) and VOSviewer (version 1.6.18).

## 3. Results

### 3.1. Scholarly Outputs and Scientific Influence

**Figure 2:**
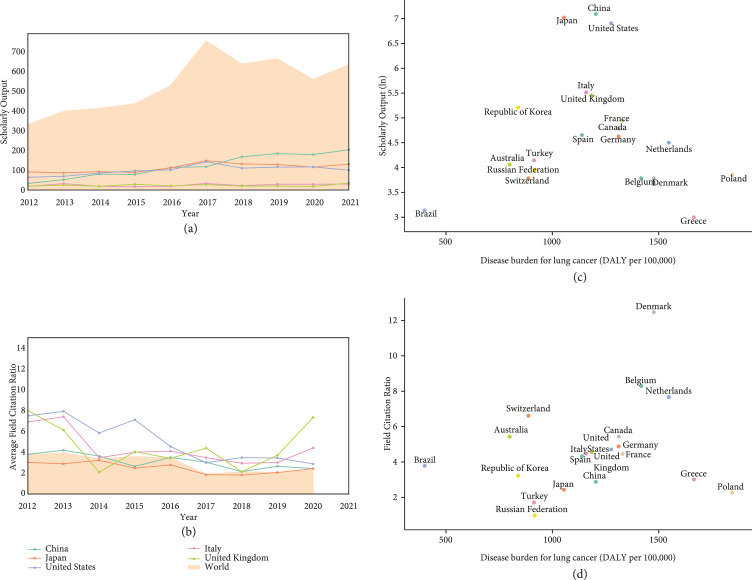
Trends of (a) annual scholarly outputs and (b) average field citation ratios in the world and top five countries in the field of lung cancer surgery, 2012-2021, and association between (c) scholarly output, (d) field citation ratio, and disease burden of lung cancer (top 20 countries in scholarly output, 2012-2021). Field citation ratios could not be obtained for publications which are less than 2 years old.

Our bibliometric research identified 5,312 publications of lung cancer surgery from Dimensions. The annual scholarly outputs and average FCRs of research related to lung cancer surgery worldwide are demonstrated in Figure 2. Overall, global scholarly outputs had almost doubled during the recent decade (2012-2021), with China, Japan, the United States, Italy, and the United Kingdom leading the way and accounting for over 70% (Table S1 in the Supplement Materials). The global average FCR of publications related to lung cancer surgery was 2.8, and Denmark and Belgium predominated in terms of scientific influence. Specifically, China has exhibited a remarkable increasing tendency in the volume of scholarly outputs over this time. In 2018, China overtook Japan and the United States, becoming the foremost country in the research field of lung cancer surgery on scholarly outputs, whereas the United Kingdom achieved the highest scientific influence among the top 5 countries in scholarly output since 2019. 

There is little association between scholarly outputs of lung cancer surgery research and disease burden of lung cancer. Countries with middle disease burden of lung cancer appeared to generate more scholarly outputs in the field of lung cancer surgery, especially in China, Japan, and the United States. However, a positive association was observed between scientific influence (FCR) and disease burden of lung cancer.

### 3.2. Cooperation Network

Distribution of cooperation types of research in the field of lung cancer surgery worldwide is demonstrated in Table 1. In the field of lung cancer surgery, nearly half of publications were under national cooperation, and about 21.0% publications were under international cooperation. Among the top 20 countries of scholarly outputs, Switzerland, Belgium, and Denmark showed high proportions of international cooperation, while Japan showed the lowest proportion of international cooperation (3.4%). Among the three cooperation types, publications under international cooperation achieved the highest FCR among most countries except Denmark, Switzerland, and Greece, and publications under institutional cooperation showed the lowest FCR among most countries. 

**Table 1:** Scholarly output and average field citation ratio of lung cancer surgery research in the top 20 countries in scholarly output by cooperation type, 2012-2021.

Country	Total		Institutional		National		International	
	Scholarly output	FCR	Scholarly output	FCR	Scholarly output	FCR	Scholarly output	FCR
World	5312	2.8	2037 (38.3%)	2.1	2162 (40.7%)	3.6	1113 (21.0%)	2.5
China	1203	2.9	467 (38.8%)	1.9	616 (51.2%)	3.0	120 (10.0%)	6.7
Japan	1120	2.4	687 (61.3%)	1.8	395 (35.3%)	3.3	38 (3.4%)	5.9
United States	998	4.7	407 (40.8%)	3.3	426 (42.7%)	4.6	165 (16.5%)	8.9
Italy	248	4.5	69 (27.8%)	2.6	109 (44.0%)	3.3	70 (28.2%)	8.4
United Kingdom	233	4.6	75 (32.2%)	1.2	86 (36.9%)	3.7	72 (30.9%)	9.7
Republic of Korea	183	3.2	49 (26.8%)	1.4	114 (62.3%)	3.5	20 (10.9%)	7.0
France	141	4.4	22 (15.6%)	2.3	73 (51.8%)	3.3	46 (32.6%)	7.5
Canada	120	5.4	24 (20.0%)	3.0	49 (40.8%)	2.1	47 (39.2%)	9.8
Spain	105	4.3	23 (21.9%)	0.8	35 (33.3%)	4.0	47 (44.8%)	6.5
Germany	102	4.9	23 (22.5%)	1.5	36 (35.3%)	2.4	43 (42.2%)	9.1
Netherlands	90	7.7	12 (13.3%)	3.7	48 (53.3%)	3.6	30 (33.3%)	14.5
Turkey	63	1.7	31 (49.2%)	1.9	24 (38.1%)	0.7	8 (12.7%)	3.9
Australia	58	5.4	18 (31.0%)	2.6	15 (25.9%)	5.2	25 (43.1%)	7.3
Russian Federation	52	1.0	21 (40.4%)	0.3	22 (42.3%)	0.2	9 (17.3%)	3.5
Poland	47	2.3	20 (42.6%)	1.4	19 (40.4%)	2.6	8 (17.0%)	3.8
Denmark	44	12.5	7 (15.9%)	8.0	15 (34.1%)	16.6	22 (50.0%)	11.0
Belgium	44	8.3	13 (29.5%)	1.5	7 (15.9%)	8.2	24 (54.5%)	11.6
Switzerland	44	6.6	6 (13.6%)	2.7	7 (15.9%)	14.7	31 (70.5%)	6.0
Brazil	23	3.8	4 (17.4%)	1.7	8 (34.8%)	0.1	11 (47.8%)	6.1
Greece	20	3.0	9 (45.0%)	2.1	8 (40.0%)	4.6	3 (15.0%)	1.8

**Figure 3:**
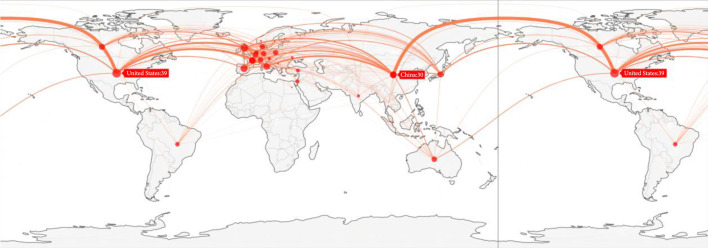
Cooperation network in the field of lung cancer surgery, 2012-2021. A point refers to a country, and its size is calculated as the numbers of cooperation partnerships of this country; a line refers to a cooperation link between two countries and the link strength is calculated as the numbers of publications under cooperation of the two countries.

The cooperation network in the field of lung cancer surgery is presented in Figure 3 and Figure S2. Based on scholarly outputs and number of partnerships, the United States, China, and Europe were the prominent centers of cooperation network, with the leading research organizations as Rigshospitalet (Denmark), St James’s University Hospital (United Kingdom), and Azienda Ospedaliera di Perugia (Italy). Besides, cooperation link between the United States and China dominated the cooperation network in terms of scholarly outputs. However, compared with China, the United States appeared to have more robust cooperation with European countries. In the cooperation network, cooperation between Canada and United States yielded the highest scientific influence measured by FCR (Canada-United States:14.1), followed by cooperation between China and Spain (China-Spain:13.8) (Table S2 in the Supplementary Materials). 

### 3.3. Emerging Research Hotspots

The distribution of research areas based on RCDC category is demonstrated in Figure S1 (in the Supplementary Materials). Research focusing on prevention, biomedical imaging, rehabilitation, and genetics dominated the field of lung cancer surgery. In terms of scientific influence of publications, research on behavioral and social science led the way with an FCR of 6.2, followed by rehabilitation (5.9) and health services (5.6). 

**Figure 4:**
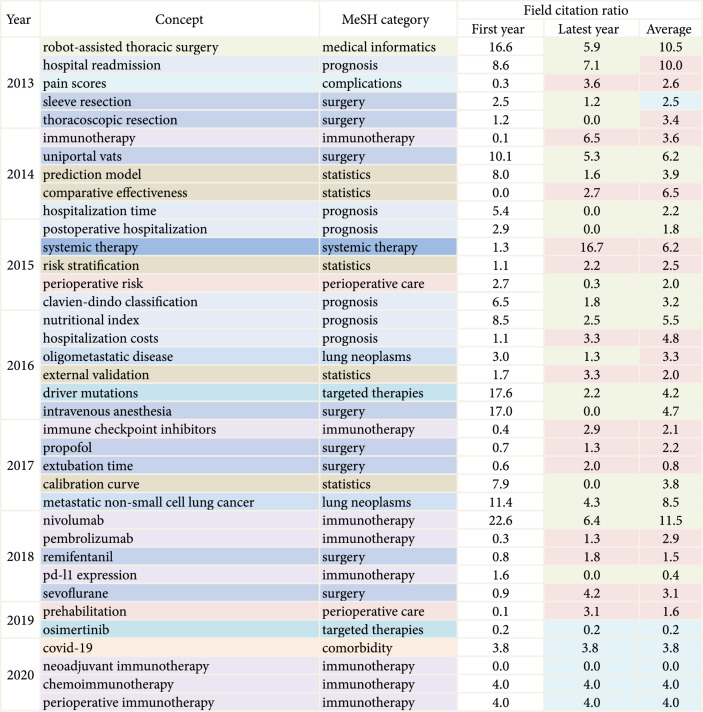
Annual emerging research concepts in the field of lung cancer surgery, 2012-2021. MeSH, Medical Subject Headings system. In terms of field citation ratio (FCR), a red square indicates that there is an increase in FCR compared to the first emerging year of a concept, a green square indicates that there is a decrease in FCR, and a blue square indicates that its FCR equals to that of the first emerging year of a concept.

Figure 4 demonstrates annual emerging research hotspots in the field of lung cancer surgery, and the cooccurrence network of emerging concepts is shown in Figure S3. In the recent decade, the major direction of innovation in the field of lung cancer surgery was immunotherapy, more specifically, neoadjuvant immunotherapy, chemoimmunotherapy, and perioperative immunotherapy. Besides, research on immunotherapy showed a considerable increase in FCR in the latest year. Systemic therapy and immune-related adverse events (irAEs) received high focus in the cooccurrence network of immunotherapy. Research on nivolumab, robot-assisted thoracic surgery, and hospital readmission achieved an average FCR of 11.5, 10.5, and 10.0, respectively, leading the way in FCR among all emerging research concepts. 

## 4. Discussion

To our knowledge, this is the first bibliometric study providing a global research landscape of lung cancer surgery, focusing on the distribution of scientific influence, cooperation network, and topic transformation of research. The comprehensive analysis of research related to lung cancer surgery, an important subfield of lung cancer, was expected to provide insights for innovative research and effective policymaking in lung cancer in the future. Specifically, this study could benefit the experts in lung cancer surgery and their future research in two ways. First, our study contributes objective evidence to a systematic understanding and assessment of the changing research trends in the field of lung cancer surgery, which helps researchers and policymakers to identify the strengths and weaknesses of the existing research and further pay more attention to the undeveloped research areas or the underlying mechanisms of the changing trend. Besides, the research overview of the lung cancer surgery field could provide references to the research on other cancers. Second, the spatial heterogeneity in the distribution and cooperation network of lung cancer surgery research depicted in this study indicates insufficient research and international cooperation on lung cancer surgery in low- and middle-income countries, and this promotes researchers conducting more studies among populations in undeveloped regions. Also, the strong association between cooperation and research quality encourages the research patterns of lung cancer surgery to be more cooperation-centered.

### 4.1. Scholarly Outputs and Scientific Influence

We found that global scholarly outputs addressing lung cancer surgery have almost doubled during the recent decade. This is consistent with the constant increase of disease burden of lung cancer, from 1.51% to 1.81% during 2010-2019 [2]. China, one of the major contributors to the increase of scholarly outputs, also experienced a remarkable surge of lung cancer burden in recent years [22], due to tobacco consumption and air pollution [23, 24]. However, viewing globally, our results suggested that the positive correlation between scholarly outputs related to lung cancer surgery and disease burden of lung cancer was not as strong as seen in China. Some studies have revealed that lung cancer was underresearched in relation to its disease burden in some European countries, and more funds were needed to support research in the field of lung cancer [25]. Furthermore, contrast to the increasing trend of global scholarly outputs in lung cancer surgery in this study, the volume of lung cancer-related research decreased during the recent decade [26]. This suggested that surgery-related research has gradually attracted more attention over the latest period. Some Eastern Asian countries, typically China, had a high scholarly output in the field of lung cancer surgery, but a relatively low overall scientific influence globally. This might be partly explained by the special healthcare system and clinicians’ promotion system in China. In China, a patient who should have been treated in primary healthcare facilities is likely to go to a well-equipped hospital, and hence, many clinicians who would do research are too often overloaded and do not have enough time for academic research [27]. However, lots of hospitals in China have brought the number of scholarly publications and the research implemented by clinicians into their promotion mechanisms. Therefore, there is a conflict between the academic expectation laid on clinicians and the time they have, which could hinder clinicians in China from conducting high-quality research [28]. More research is needed to improve the situation. 

### 4.2. Cooperation Network

In this study, a global network of cooperation in the field of lung cancer surgery was exhibited and suggested that countries that contributed more towards international collaboration were the United States, China, and Europe. During 2004-2013, a higher proportion of international cooperation on lung cancer was observed in European countries [4], which was consistent with our results. Our results illustrated China and United States have the highest number of partner countries as well. However, collaboration between other Asian countries remained to be strengthened. Active collaboration is usually found to be implemented between developed countries and more likely to produce high-quality research outputs. The participation in international cooperation of lower middle-income countries is limited, needing more support from developed countries [29, 30]. In addition, this study revealed a strong collaboration network between China and the United States. Not only in the field of lung cancer surgery, similar trends were also reported in immunotherapy that the collaboration relationship between China and the United States was the strongest [30]. The intense United States-China cooperation may be explained by their strong basic science research capability, comprehensive research resources, and great investment. 

### 4.3. Emerging Research Hotspots

There was wide variability in scholarly outputs and scientific influence among different research areas in the field of lung cancer surgery. Contrary to our hypothesis, research on prevention and biomedical imaging dominated in the field of lung cancer surgery, while basic science research was not a popular research category. Besides, research on behavioral and social science received the highest scientific influence. These results may indicate that research related to lung cancer surgery, rather than basic research, was more patient-centered, specifically focusing on prevention, rehabilitation, and behavioral and social science. Compared to the research modality of general cancer research in the recent decade, research of lung cancer surgery paid more attention to prevention, while general cancer research focused more on cell biology [10]. The possible reason behind was that surgical complications and surgery-related mortality were common and important issues to be addressed in the field of lung cancer surgery. This was further supported by the result that surgical complications and prognosis have obtained high attention in the emerging research topics in the field of lung cancer surgery. 

Consistent with the research hotspots of lung cancer before the recent decade [4], genetics remains a key issue, and immunotherapy was leading the innovation direction in the field of lung cancer surgery. Research on immunotherapy showed a considerable increase in scientific influence in recent years, but the scholarly outputs of immunotherapy were still limited in research related to lung cancer surgery. Before 2011, surgery combined with chemotherapy or radiotherapy was the main treatment for patients with lung cancer [31, 32]. In 2004, the discovery of epidermal growth factor receptor (EGFR) mutations that confer sensitivity to tyrosine kinase inhibitors (TKI) in lung adenocarcinomas heralded the beginning of the era of precision medicine for lung cancer [33]. Since then, the importance of commitment to genetics and immunotherapy is increasing in the era of precision medicine, which was consistent with the innovative direction of research found in this study. Studies have suggested that compared to the traditional chemotherapy, precision oncology based on genetic therapy and immunotherapy has helped improve treatment outcomes and quality of life for patients with lung cancer [34, 35]. For instance, in patients with advanced non-small-cell lung cancer (NSCLC), significant improvement has been observed in survival results with monoclonal antibody drugs compared to chemotherapy [36, 37]. In the recent decade, immunotherapy has gradually been implemented in second-line treatment and then first-line treatment [32, 38, 39]. 

Our results suggested that the innovation research interests in immunotherapy focused on neoadjuvant immunotherapy, chemoimmunotherapy, and perioperative immunotherapy. The promising performance of immunotherapy for lung cancer, combined with the success of immunotherapy in the neoadjuvant setting for several cancers, such as melanoma [40], breast cancer [41], and urological cancer [42], contributed to a strong rationale for pursuing the neoadjuvant immunotherapy studies in lung cancer. More recently, the use of immunotherapy for the adjuvant treatment of patients with surgically resectable lung cancer has increasingly become an effective but controversial treatment [43]. Current studies have preliminarily confirmed that the neoadjuvant immunotherapy could attain effective pathological remission [44], prolonged survival time [45], and improved effectiveness and safety of the treatment [46]. However, many questions regarding the use of neoadjuvant immunotherapy for lung cancer, such as its target population, standards for choice of immune drugs and doses, and the pharmacological effects [43, 47], remain to be answered. Specifically, the increasing research attention on irAEs further emphasizes the continuous concerns about the safety of neoadjuvant immunotherapy. More studies are needed to further investigate the optimal use and health effect of neoadjuvant immunotherapy on patients with lung cancer. 

There are several limitations of the present study. First, we selected publications available in the Dimensions database for analysis, while some publications in national language journals have not been included. This may cause a bias against scholarly outputs from non-English speaking countries. Second, we excluded publications from journals with low scholarly outputs related to lung cancer surgery to ensure quality of publications for analysis. This could lead to selection bias to some extent in our study. Third, the RCDC category and research concepts of each publication were automatically derived from the publication text by machine learning methods. Therefore, it is possible that some publications were miscategorized. Besides, some categories in RCDC system were quite general, while more specific research category was not available in Dimensions. The RCDC system may not be compatible enough for the field of lung cancer surgery. Furthermore, some specific metrics of publications, such as the societal impact, were not evaluated in this analysis because of limited information available in Dimensions.

## 5. Conclusions

Our findings have established a comprehensive global scientific landscape of lung cancer surgery research in the recent decade. It suggested a growing participation across the world in lung cancer surgery research and an international cooperation research network dominated by the United States, China, and Europe. Research on immunotherapy led the direction of innovation in lung cancer surgery research and showed a promising scientific influence in the recent decade. The study findings are expected to provide researchers and policymakers with interesting insights into lung cancer research and further generate evidence to support decision-making in improving prognosis for patients with lung cancer.

## Data Availability

PL and JZ had full access to all the data in the study and take responsibility for the integrity of the data and the accuracy of the data analysis. This paper was written using data obtained on DATE, from Digital Science’s Dimensions platform, available at https://app.dimensions.ai (accessed on August 31, 2022). Access was granted to subscription-only data sources under license agreement.

## Supplementary Materials

Supplementary Materials Text S1: the method of concept identification in dimensions. Figure S1: scholarly output and average FCR in each RCDC category in the field of lung cancer surgery, 2012-2021. Figure S2: international cooperation network of research organizations in the field of lung cancer surgery, 2012-2021. Figure S3: cooccurrence network of emerging concepts in the field of lung cancer surgery, 2012-2021. Table S1: annual scholarly outputs and average field citation ratios in the field of lung cancer surgery in the top 20 countries in scholarly output, 2012-2021. Table S2: major cooperation partnerships of top five countries in scholarly output under international cooperation, 2012-2021.
