# Supplementary material for: Research Trends and Emerging Hotspots of Lung Cancer Surgery during 2012-2021: A 10-Year Bibliometric and Network Analysis
Source: Health Data Sci. 2022 Oct 20;2022:9797842. doi: 10.34133/2022/9797842 (PMC10880176; doi:10.34133/2022/9797842)
Supplement: Supplementary Materials — Text S1: the method of concept identification in dimensions. Figure S1: scholarly output and average FCR in each RCDC category in the field of lung cancer surgery, 2012-2021. Figure S2: international cooperation network of research organizations in the field of lung cancer surgery, 2012-2021. Figure S3: cooccurrence network of emerging concepts in the field of lung cancer surgery, 2012-2021. Table S1: annual scholarly outputs and average field citation ratios in the field of lung cancer surgery in the top 20 countries in scholarly output, 2012-2021. Table S2: major cooperation partnerships of top five countries in scholarly output under international cooperation, 2012-2021. [file 9797842.f1.docx]

Supplementary Materials

**Page 2: Supplement Texts S1**

Text S1. The method of concept identification in Dimensions

**Page 3-5: Supplement Figures S1-3**

Figure S1. Scholarly output and average FCR in each RCDC category in the field of lung cancer surgery, 2012-2021.

Figure S2. International cooperation network of research organizations in the field of lung cancer surgery, 2012-2021.

Figure S3. Cooccurrence network of emerging concepts in the field of lung cancer surgery, 2012-2021.

**Page 6-7: Supplemental Tables S1-2**

Table S1. Annual scholarly outputs and average field citation ratios in the field of lung cancer surgery in the top 20 countries in scholarly output, 2012-2021.

Table S2. Major cooperation partnerships of top five countries in scholarly output under international cooperation, 2012-2021.

Supplemental Texts

S1 The method of concept identification in Dimensions

In Dimensions, standardized and reproducible concepts of papers were identified algorithmically using a machine learning approach, based on classifications carried out by experts in each system, and a very good match compared to manual coding was claimed by the Dimensions team. Specifically, Dimensions has the capacity for an expert to build a classification based on a set of search terms. An expert starts the process by searching for a general term or a longer constructed search string to amass an inclusive set of objects that fall into the presumptive category. Concepts are extracted from the corpus that has been returned and the expert has the opportunity to boost particular keywords, re-ranking the search results to produce a different relevance score, or exclude objects that include particular terms. By repeating this process, the expert (who is an expert in the subject but not an expert in computer coding) can define a field in a way that a computer is able to understand (Hook et al., 2018).

References

Hook, D. W., S. J. Porter and C. Herzog (2018). "Dimensions: Building Context for Search and Evaluation." Frontiers in Research Metrics and Analytics 3: 23.

Supplemental Figures


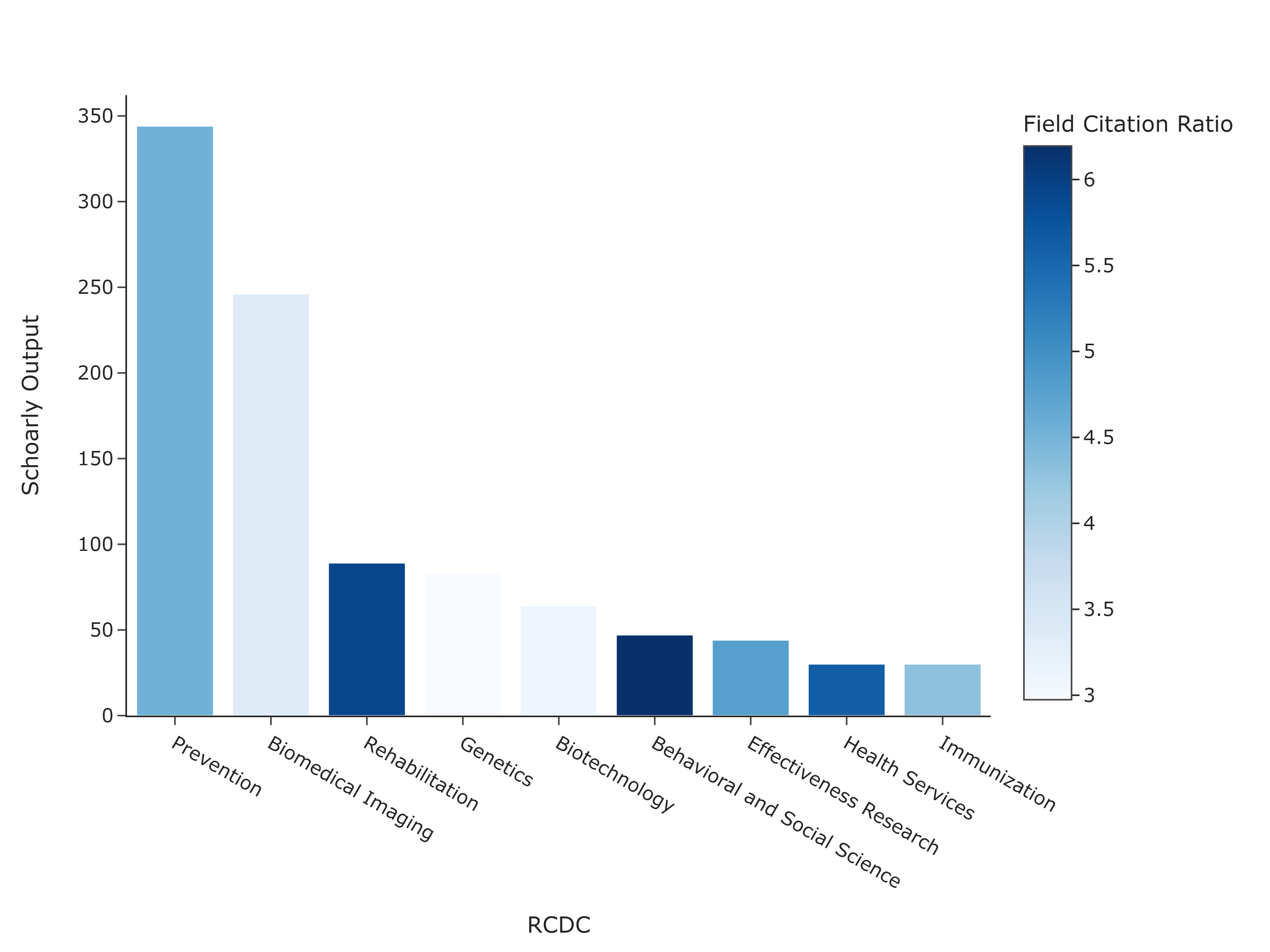


Figure S1. Scholarly output and average FCR in each RCDC category in the field of lung cancer surgery, 2012-2021. FCR, Field Citation Ratio; RCDC, Research, Condition, and Disease Categorization system.


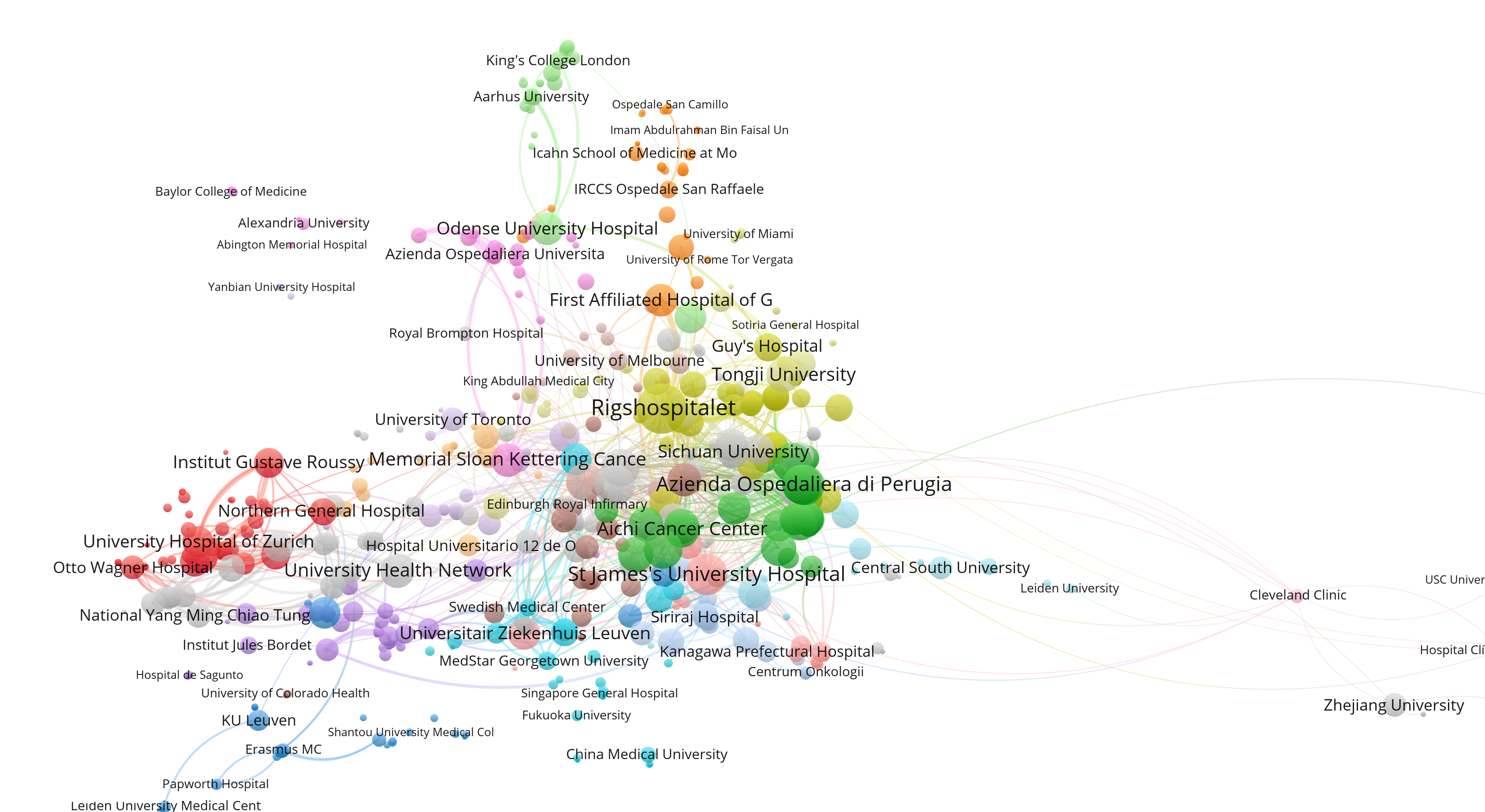


Figure S2. International cooperation network of research organizations in the field of lung cancer surgery, 2012-2021. A point refers to a research organization and its size is calculated as the numbers of cooperation partnerships of this organization; a line refers to a cooperation link between two research organizations and the link strength is calculated as the scholarly output under cooperation of the two organizations.


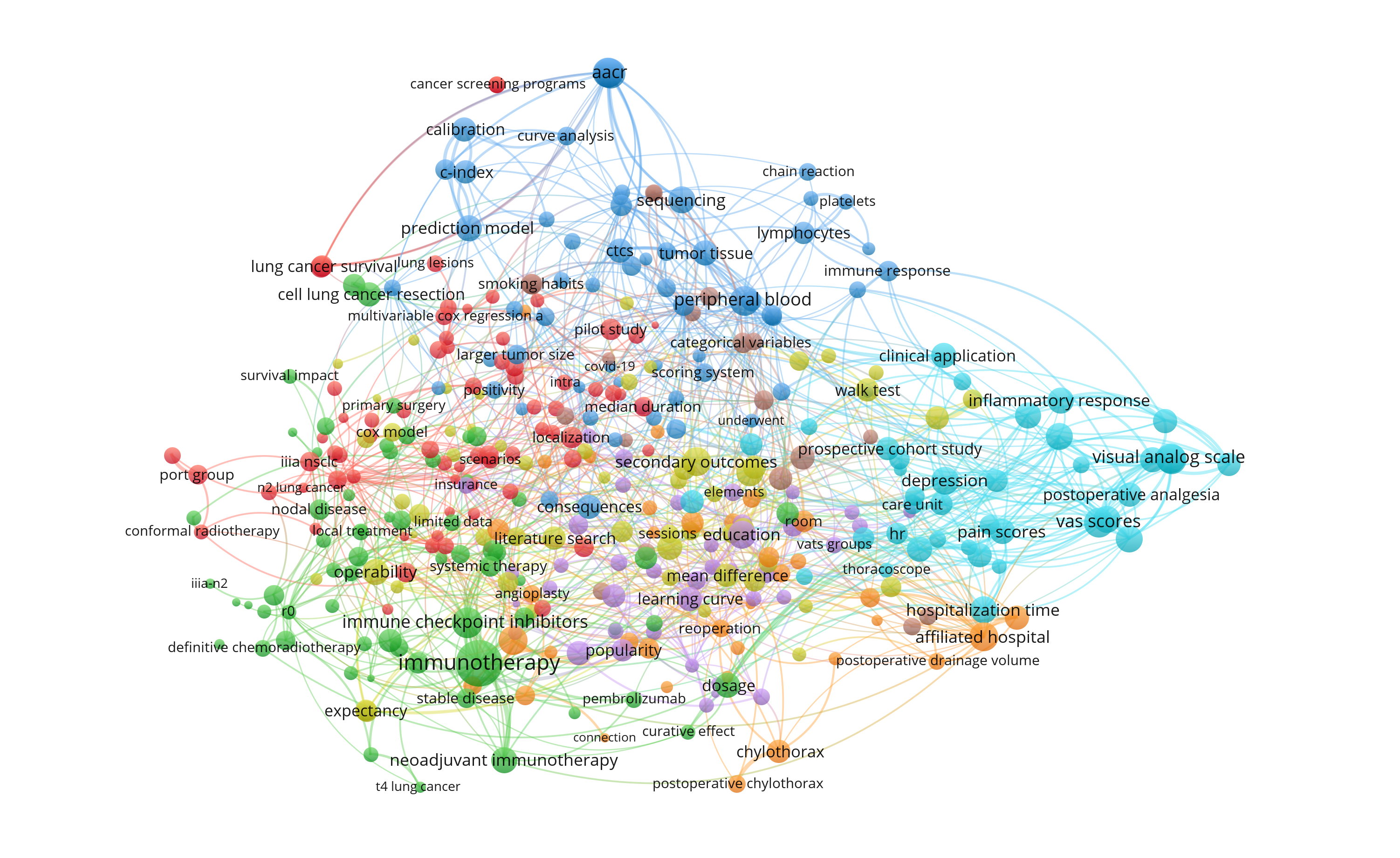


Figure S3. Cooccurrence network of emerging concepts in the field of lung cancer surgery, 2012-2021. A point refers to a concept and its size is calculated as its frequency; a line refers to a cooccurrence link between two concepts and the link strength is calculated as their cooccurrence frequency.

Supplemental Tables

Table S1. Annual scholarly outputs and average field citation ratios in the field of lung cancer surgery in the top 20 countries in scholarly output, 2012-2021. Data was presented like *annual scholarly output (average field citation ratio)*.

| Country | 2012 | 2013 | 2014 | 2015 | 2016 | 2017 | 2018 | 2019 | 2020 | 2021^a^ | Total |
| --- | --- | --- | --- | --- | --- | --- | --- | --- | --- | --- | --- |
| World | 328 (3.8) | 395 (3.9) | 410 (3.4) | 433 (3.6) | 524 (3.5) | 747 (1.9) | 633 (2.1) | 658 (2.1) | 555 (2.3) | 629 (-) | 5312 (2.8) |
| China | 33 (3.8) | 52 (4.2) | 80 (3.6) | 78 (2.7) | 113 (3.5) | 117 (3.1) | 167 (2.2) | 183 (2.7) | 178 (2.4) | 202 (-) | 1203 (2.9) |
| Japan | 91 (3.0) | 86 (2.9) | 92 (3.2) | 89 (2.5) | 111 (2.8) | 147 (1.9) | 131 (1.8) | 128 (2.1) | 115 (2.4) | 130 (-) | 1120 (2.4) |
| United States | 64 (7.5) | 69 (7.9) | 85 (5.9) | 96 (7.1) | 101 (4.6) | 142 (3.0) | 110 (3.5) | 115 (3.5) | 116 (2.9) | 100 (-) | 998 (4.7) |
| Italy | 18 (6.9) | 32 (7.4) | 19 (3.5) | 17 (4.0) | 19 (4.1) | 33 (3.5) | 22 (3.0) | 29 (3.0) | 29 (4.4) | 30 (-) | 248 (4.5) |
| United Kingdom | 19 (8.0) | 25 (6.1) | 19 (2.1) | 29 (4.1) | 21 (3.4) | 27 (4.4) | 20 (2.1) | 20 (3.7) | 18 (7.4) | 35 (-) | 233 (4.6) |
| Republic of Korea | 7 (2.0) | 16 (5.1) | 16 (2.6) | 21 (4.4) | 17 (2.6) | 18 (1.8) | 17 (3.1) | 29 (2.5) | 23 (4.2) | 19 (-) | 183 (3.2) |
| France | 9 (5.2) | 18 (3.1) | 8 (6.8) | 9 (12.0) | 13 (3.5) | 15 (2.4) | 15 (3.1) | 11 (3.6) | 19 (4.7) | 24 (-) | 141 (4.4) |
| Canada | 12 (10.8) | 18 (7.0) | 8 (2.1) | 10 (3.2) | 7 (2.3) | 15 (2.9) | 10 (4.3) | 11 (5.4) | 7 (9.8) | 22 (-) | 120 (5.4) |
| Spain | 3 (1.1) | 10 (2.6) | 7 (5.7) | 13 (1.9) | 13 (8.6) | 6 (2.8) | 16 (2.1) | 17 (3.5) | 7 (14.0) | 13 (-) | 105 (4.3) |
| Germany | 6 (5.0) | 8 (1.9) | 9 (1.4) | 16 (9.5) | 6 (2.1) | 7 (5.7) | 14 (2.9) | 13 (5.1) | 11 (6.1) | 12 (-) | 102 (4.9) |
| Netherlands | 9 (6.2) | 9 (7.9) | 4 (2.2) | 6 (38.9) | 7 (4.5) | 8 (4.8) | 12 (6.7) | 10 (3.2) | 13 (3.7) | 12 (-) | 90 (7.7) |
| Turkey | 3 (4.5) | 2 (3.6) | 4 (1.1) | 6 (0.9) | 8 (3.6) | 10 (0.8) | 10 (2.1) | 10 (0.6) | 7 (1.3) | 3 (-) | 63 (1.7) |
| Australia | 1 (35.4) | 3 (8.5) | 7 (5.4) | 5 (6.7) | 5 (12.4) | 8 (0.6) | 7 (2.9) | 11 (1.2) | 4 (13.8) | 7 (-) | 58 (5.4) |
| Russian Federation | 2 (0.0) | 1 (0.0) | 4 (1.6) | 2 (0.6) | 5 (0.0) | 7 (2.2) | 4 (1.4) | 6 (0.3) | 11 (1.1) | 10 (-) | 52 (1.0) |
| Poland | 0 (-) | 4 (3.1) | 8 (1.1) | 4 (0.7) | 5 (2.3) | 5 (2.7) | 7 (2.1) | 8 (3.1) | 4 (3.5) | 2 (-) | 47 (2.3) |
| Denmark | 6 (5.1) | 6 (15.9) | 5 (2.2) | 5 (27.9) | 6 (29.7) | 3 (2.4) | 4 (4.3) | 5 (8.0) | 2 (2.3) | 2 (-) | 44 (12.5) |
| Belgium | 2 (3.8) | 7 (7.1) | 5 (4.8) | 4 (26.5) | 5 (3.4) | 6 (4.0) | 1 (11.8) | 6 (4.4) | 5 (14.6) | 3 (-) | 44 (8.3) |
| Switzerland | 0 (-) | 6 (1.6) | 1 (1.2) | 3 (1.5) | 6 (7.6) | 3 (7.2) | 5 (3.5) | 3 (5.1) | 5 (18.3) | 12 (-) | 44 (6.6) |
| Brazil | 0 (-) | 0 (-) | 3 (2.4) | 0 (-) | 2 (7.7) | 3 (3.5) | 3 (0.5) | 5 (5.5) | 0 (-) | 7 (-) | 23 (3.8) |
| Greece | 0 (-) | 1 (11.7) | 8 (3.8) | 2 (1.1) | 3 (1.5) | 1 (0.0) | 0 (-) | 1 (1.2) | 2 (2.1) | 2 (-) | 20 (3.0) |

Table S2. Major cooperation partnerships of top five countries in scholarly output under international cooperation, 2012-2021.

| Countries | Major partnership | Scholarly output | FCR |
| --- | --- | --- | --- |
| China | United States | 71 (5.9%) | 7.1 |
|  | Italy | 34 (2.8%) | 8.6 |
|  | United Kingdom | 26 (2.2%) | 10.5 |
|  | Japan | 21 (1.7%) | 10.7 |
|  | Spain | 21 (1.7%) | 13.8 |
| Japan | China | 21 (1.9%) | 10.7 |
|  | United States | 21 (1.9%) | 8.4 |
|  | Italy | 13 (1.2%) | 10.1 |
|  | United Kingdom | 12 (1.1%) | 10.4 |
|  | Canada | 9 (0.8%) | 12.8 |
| United States | China | 71 (7.1%) | 7.1 |
|  | Italy | 36 (3.6%) | 12.4 |
|  | Canada | 26 (2.6%) | 14.1 |
|  | United Kingdom | 24 (2.4%) | 12.2 |
|  | Japan | 21 (2.1%) | 8.4 |
| Italy | United States | 36 (14.5%) | 12.4 |
|  | China | 34 (13.7%) | 8.6 |
|  | United Kingdom | 21 (8.5%) | 6.7 |
|  | Germany | 18 (7.3%) | 4.1 |
|  | France | 17 (6.9%) | 6.0 |
| United Kingdom | China | 26 (11.2%) | 10.5 |
|  | United States | 24 (10.3%) | 12.2 |
|  | Italy | 21 (9.0%) | 6.7 |
|  | Germany | 20 (8.6%) | 11.1 |
|  | France | 19 (8.2%) | 12.7 |
